# Genetic and Environmental Influences on Infant Growth: Prospective Analysis of the Gemini Twin Birth Cohort

**DOI:** 10.1371/journal.pone.0019918

**Published:** 2011-05-27

**Authors:** Laura Johnson, Clare H. Llewellyn, Cornelia H. M. van Jaarsveld, Tim J. Cole, Jane Wardle

**Affiliations:** 1 Cancer Research UK Health Behavior Research Centre, Department of Epidemiology and Public Health, University College London, London, United Kingdom; 2 Department of Public Health and Primary Care, University of Cambridge, Cambridge, United Kingdom; 3 MRC Centre of Epidemiology for Child Health, Institute of Child Health, University College London, London, United Kingdom; Genentech Inc., United States of America

## Abstract

**Objective:**

Infancy is a critical period during which rapid growth potentially programs future disease risk. Identifying the modifiable determinants of growth is therefore important. To capture the complexity of infant growth, we modeled growth trajectories from birth to six months in order to compare the genetic and environmental influences on growth trajectory parameters with single time-point measures at birth, three and six months of age.

**Methods:**

Data were from Gemini, a population sample of 2402 UK families with twins. An average 10 weight measurements per child made by health professionals were available over the first six months. Weights at birth, three and six months were identified. Longitudinal growth trajectories were modeled using SITAR utilizing all available weight measures for each child. SITAR generates three parameters: size (characterizing mean weight throughout infancy), tempo (indicating age at peak weight velocity (PWV)), and velocity (reflecting the size of PWV). Genetic and environmental influences were estimated using quantitative genetic analysis.

**Results:**

In line with previous studies, heritability of weight at birth and three months was low (38%), but it was higher at six months (62%). Heritability of the growth trajectory parameters was high for size (69%) and velocity (57%), but low (35%) for tempo. Common environmental influences predominated for tempo (42%).

**Conclusion:**

Modeled growth parameters using SITAR indicated that size and velocity were primarily under genetic influence but tempo was predominantly environmentally determined. These results emphasize the importance of identifying specific modifiable environmental determinants of the timing of peak infant growth.

## Introduction

Infancy is a critical period during which growth patterns may program lifelong risk of obesity and chronic disease [1], [2], [3], [4], [5]. Normal infant growth consists of an initial fall in weight after birth followed by increasing weight gain to a peak at six weeks (peak weight velocity (PWV)) after which the rate of weight gain declines to a plateau around six months [6]. Both weight velocity and age at PWV (an indicator of growth tempo) vary between infants. Similar to other tempo indicators (e.g. adiposity rebound, puberty onset, peak height velocity), earlier PWV is associated with a higher risk of adult disease [2], [7], [8], [9]; suggesting that an accelerated tempo of development is detrimental to longer-term health. Rapid weight gain is a well-studied risk factor for obesity [10], but weight gain based on just two weight measurements cannot estimate age at PWV or characterize variations in velocity throughout infancy, which may be crucial to the development of chronic disease.

Quantitative genetic studies of single time-point weight measures between birth and three months indicate that variation in weight is primarily attributable to the environment; with just 10–44% explained by genetic influences [11], [12], [13], [14], [15], [16], [17], [18], whereas from five months onwards genes play a larger role (66–90% heritability) [13], [18], [19]. A summary of growth velocity can be captured by using repeated measures of weight throughout infancy and mathematically modeling growth trajectories using one of a range of infant growth curve models [20], [21], [22], [23]. Two studies modeled infant growth velocity (using a polynomial of degree 4 model or the Count model) based on an average of 12 weight measures between birth and 2.5 years in 681 children from 169 families [24], and in a large sample of twins (n = 3477 pairs) [22]. The heritability of growth velocity was estimated as 28% in the family study but 63% in the twin sample [22], [24].

No studies to date have characterized the heritability of the timing of PWV, nor has any study established whether the heritability of modeled growth parameters, which may be more reliable because they use data from multiple time-points, is higher than single time-point measures of weight. SuperImposition by Translation And Rotation (SITAR) is a novel method of modeling growth that estimates three parameters: size, velocity and tempo (equivalent to age at PWV) [25]. In this study we assessed the contribution of genetic and environmental factors to single measurements of weight at birth, three and six months and the three SITAR growth parameters in a large twin birth cohort.

## Methods

### Ethics Statement

Parents provided informed written consent for their family to participate in the study and ethical approval was granted by the University College London Committee for the Ethics of non–National Health Service Human Research. All aspects of data collection and storage were in accordance with the standards stipulated by this body.

Data came from Gemini [26], a birth cohort initiated in 2007 to investigate genetic and environmental influences on appetite, activity and growth from birth to 5 years. All families in England and Wales with live twin births between March and December 2007 (n = 6754) were eligible for recruitment and were asked by the Office of National Statistics for consent to be contacted by the research team. A total of 3435 families (51%) agreed to be contacted, of whom 2402 (70% of those contacted and 39% of all eligible families) returned a baseline questionnaire. Sex and gestational age were maternally reported. A validated questionnaire [27] established the zygosity of same-sex twin pairs as monozygotic (MZ) or dizygotic (DZ) (opposite-sex twins are all DZ). Children in England and Wales are measured regularly from birth by health professionals and the values are recorded in a personal health record kept by the parents. Parents were asked to photocopy the relevant pages of their children's health records or transcribe the measurements into the questionnaire. Parents returned the weight data when the twins were on average 8 (SD 2) months old, with a median of 10 (Inter-Quartile Range (IQR) 7) weight measurements per child recorded between birth and a median of 6.6 (IQR 3.1) months old.

### Statistical analyses

Continuous variables are described between twin pairs by calculating the sample mean and SD of the means within each twin pair. Analogously, the within-pair SD was calculated as the SD of the difference within each twin pair. Categorical variables are described with frequencies and percentages. Descriptive analyses were performed in SPSS v15 (SPSS Inc, Chicago, IL).

#### Infant growth modeling

**Figure 1:**
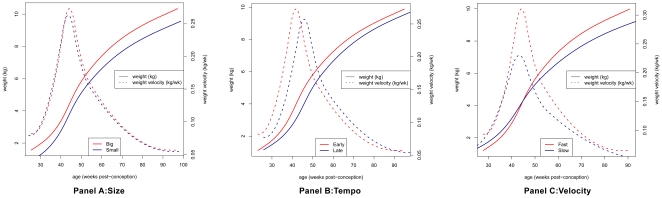
Average weight and weight velocity curves for extremes^a^ of size (A), tempo (B) and velocity (C) in infancy. ^a^ Extreme groups were based on tertiles (T1, T2, T3) of SITAR parameters such that ‘small’ children were in T1 for size and T2 for tempo and velocity and ‘big’ children were in T3 for size and T2 for tempo and velocity; ‘early’ children were in T1 for tempo and T2 for size and velocity and ‘late’ children were in T3 for tempo and T2 for size and velocity; ‘slow’ children were in T1 for velocity and T2 for tempo and size and ‘fast’ children were in T3 for velocity and T2 for tempo and size.

Weight growth curves were analyzed using the SuperImposition by Translation and Rotation (SITAR) method [25]. SITAR is a shape invariant model with random effects, originally used for infant growth modeling by Beath [28]. It involves the estimation of an average growth curve for the sample, plus a set of three parameters for each individual that together transform the average curve to match the individual's growth. The average curve is fitted as a cubic spline and individual parameters are estimated as mixed-model random effects based on at least one weight measure per child. Growth curves for children with fewer measures of weight are therefore ‘shrunk’ towards the average curve because of the reduced information. S*ize* is an up/down shift of the average curve, indicating whether the infant is bigger or smaller than average (see figure 1, panel A). *Tempo* is a left/right shift of the average curve, indicating whether the timing of PWV is earlier or later than average (figure 1, panel B). *Velocity* is a shrinking/stretching of the age scale, because shrinking the age scale makes the curve steeper (increasing velocity) and stretching the scale creates a shallower slope (reducing velocity) - effectively this is a rotation of the growth curve indicating the rate at which ‘growth time’ passes for an individual relative to the average (figure 1, panel C). The SITAR analysis was done using the nlme library [29] in the statistical package R [30].

Separate SITAR models were fitted for first- and second-born twins, and the parameter estimates were compared within twin pairs. The modeling involved weight in kg and postmenstrual age (weeks). Size was measured in kg, tempo in weeks and velocity in fractional units, which multiplied by 100 correspond to a percentage of mean velocity [31]. Negative values represent smaller/earlier/slower and positive values larger/later/faster than average.

Single time-point measures of weight at birth, three and six months were identified for quantitative genetic analysis. Weight ‘at three months’ was defined as weight measured between two and four months closest to three months and weight ‘at six months’ was weight measured between five and seven months closest to six months (exact age was recorded). Weight standard deviation scores (SDS) at birth, three and six months were calculated adjusting for age, sex and gestational age based on the British 1990 growth reference [32], [33]. Change in weight SDS between birth and, three or six months was calculated by subtracting weight SDS at the earlier from weight SDS at the later time-point.

#### Heritability analyses

Heritability was estimated using intra-class correlation (ICC) coefficients and quantitative genetic analysis. ICCs assess similarity within and between twin pairs; a higher ICC in MZ than DZ twin pairs indicates greater genetic influence. All heritability analyses were adjusted for age and sex using the residual method, a standard practice because age and sex are perfectly correlated within same-sex pairs, which mimics and inflates common environmental variation.

Quantitative genetic analysis provides robust estimates of genetic and environmental influences by partitioning trait variation into an additive genetic component (A; which makes more genetically related children more similar), a common environment component (C; environmental factors that make children in the same family more similar) and a unique environment component (E; which makes children in the same family more different but also includes measurement error) as well as generating confidence intervals. Using maximum-likelihood structural equation modelling, trait variance is partitioned based on expected covariance structures between MZ and DZ twins using the following assumptions: 1) MZs share 100% of their DNA (so their coefficient of genetic relatedness is 1); 2) DZs share 50% of their segregating genes (so their coefficient of genetic relatedness is 0.5); 3) MZs and DZs have the same common environmental exposures (fixing the covariance of the C component is 1 for both types of twins); 4) each twin's unique environment is uncorrelated with their sibling. More constrained sub-models dropping A or C or both were examined, but in all cases the full ACE model fitted best (as judged by the Bayesian Information Criterion and change in -2LL χ^2^ tests of sub-models producing a p<0.05) so only ACE model results are presented. Sex differences in A, C and E were also investigated using a sex-limitation model, but none were significant.

All available data on each weight/growth variable were used for the genetic analyses, but single time-point data had some missing cases, which meant that the sample size varied. Estimates of size, tempo and velocity were available for 2340 twin pairs, while weight SDS data were available for 2322 twin pairs at birth, 2110 pairs at three months and 1717 pairs at six months. Data on 2099 pairs were available for SDS change from birth to three months and 1633 pairs for SDS change from birth to six months. Twin analyses were carried out using Mx Maximum-Likelihood Structural Equation Modeling Software (version 32; Virginia Commonwealth University, Richmond, VA).

## Results

Descriptive characteristics of the Gemini sample are displayed in Table 1. As expected of twins, they were born earlier and smaller than the British 1990 reference singleton children. MZ twins were also born earlier than DZ twins, which explained their lower weight at birth, three and six months. The SITAR model for first-born twins (based on 21,617 weights from 2340 individuals) had a residual SD of 0.16 kg compared with a residual SD of 0.69 kg for the population growth curve with no random effects. Thus the addition of the SITAR parameters to the model reduced the variance by 94%; providing a much better fit to the data. Results for the second-born twins were effectively the same as for the first-born. The random effect parameters had means of zero (by definition) and SDs of 0.68 kg for size, 3.7 weeks for tempo and 24% for velocity, which means that 95% of infants had sizes within 1.36 kg of mean size, tempos within 7.4 weeks of mean tempo, and velocities within 48% of mean velocity [31].

**Table 1:** Descriptive statistics of weight and weight change in infancy in the Gemini cohort.

		Monozygotic twins				Dizygotic twins			
		N (pairs)	Overall		Within pair	N (pairs)	Overall		Within pair
			Mean	(SD)	SD		Mean	(SD)	SD
Sex	Male n (%)	345 (47%)				400 (25%)			
	Female n (%)	384 (53%)				389 (24%)			
	Opposite sex n (%)					816 (51%)			
Gestational age (weeks)		727	35.6	(2.5)		1598	36.5	(2.4) b	
Age (weeks) at three month weight measurement		636	12.9	(1.5)	0.0	1421	13.0	(1.4)	0.0
Age (weeks) at six month weight measurement		515	25.6	(1.9)	0.0	1139	25.8	(1.9)	0.0
Weight (kg) at birth		707	2.34	(0.52)	0.34	1561	2.52	(0.49) b	0.41
Weight (kg) at three months		636	5.03	(0.88)	0.51	1421	5.22	(0.80) b	0.76
Weight (kg) at six months		515	6.98	(0.95)	1.03	1139	7.16	(0.89) b	1.40
Weight gain (kg) from birth to three months		634	2.69	(0.61)	0.34	1409	2.70	(0.57)	0.58
Weight gain (kg) from birth to six months		514	4.68	(0.78)	0.89	1119	4.67	(0.71)	1.27
Weight SDS at birth a		706	−0.53	(0.85)	0.93	1555	−0.57	(0.76)	1.06
Weight SDS at three months a		635	−0.28	(1.01)	0.88	1415	−0.28	(0.93)	1.10
Weight SDS at six months a		515	−0.28	(1.05)	1.22	1137	−0.25	(0.97)	1.51
Change in weight SDS from birth to three months a		633	0.26	(0.94)	0.66	1404	0.28	(0.84)	0.88
Change in weight SDS from birth to six months a		514	0.30	(1.04)	1.16	1119	0.31	(0.95)	1.43

a Standard deviation scores were calculated from weight, exact age at 3 or 6 months, gestational age and sex compared with British 1990 growth reference data [33].

b Indicates DZ is significantly different from MZ twin pairs at p<0.0001.

The size parameter was positively correlated with weight at birth, three and six months (r = 0.39, r = 0.41, r = 0.69 respectively). Velocity was associated with greater changes in weight SDS from birth to three (r = 0.68) and birth to six (r = 0.70) months. A later PWV was associated with longer gestation (r = 0.23) and lower weight SDS at birth, three and six months (r = −.022, r = −0.56, r = −0.22, respectively); conversely those who were younger at PWV tended to be larger at all ages.

ICCs and results of the quantitative twin analyses for weight at birth, three and six months, alongside SITAR growth parameters are shown in Table 2. ICCs for all measures were higher for MZ than DZ twins indicating genetic influence on infant growth; but the difference varied across growth indicators. The twin analyses indicated that heritability varied from just 38% at birth and three months to 62% at six months. Weight SDS change between birth and three months was 35% heritable, increasing to 57% for change in weight SDS from birth to six months. Unique environmental influences (which include measurement error) explained most of the variation in birth weight, while common environment effect was strongest for change in weight SDS from birth to three months (Table 2).

**Table 2:** Intra-class correlations and heritability of weight, weight change and SITAR growth parameters from birth to six months in Gemini.

	Intra-class correlations		Additive genetic (A) a	Common environmental (C) a	Unique environmental (E) a
	MZ	DZ			
Weight SDS at birth b	0.53 (0.48, 0.59)	0.34 (0.29, 0.38)	38 (25, 51)	12 (2, 22)	50 (45, 55)
Weight SDS at three months c	0.68 (0.64, 0.72)	0.47 (0.43, 0.51)	38 (28, 48)	29 (20, 37)	33 (30, 37)
Weight SDS at six months c	0.77 (0.73, 0.80)	0.47 (0.42, 0.51)	62 (53, 73)	15 (6, 24)	22 (20, 26)
Change in weight SDS from birth to three months c	0.76 (0.72, 0.79)	0.56 (0.52, 0.59)	35 (26, 44)	37 (29, 44)	28 (25, 32)
Change in weight SDS from birth to six months c	0.81 (0.77 0.83)	0.54 (0.50, 0.59)	57 (47, 67)	21 (13, 30)	22 (19, 25)
Size d	0.80 (0.77, 0.82)	0.48 (0.44, 0.52)	69 (60, 77)	11 (3, 18)	20 (18, 23)
Tempo d	0.76 (0.73, 0.79)	0.60 (0.57, 0.63)	35 (27, 42)	42 (35, 48)	24 (21, 27)
Velocity d	0.84 (0.82, 0.86)	0.49 (0.45, 0.53)	57 (50, 65)	26 (19, 32)	17 (15, 19)

a % of variation (95%CI) estimated from standard ACE model-fitting analyses to model heritability of continuous data.

b Adjustments to scores: scores modeled were residuals adjusted for gestational age and sex.

c Adjustments to scores: scores modeled were residuals adjusted for gestational age, exact age at 3 or 6 months and sex.

d Adjustments to scores: scores modeled were residuals adjusted for sex.

The heritability of the SITAR growth parameters was moderate for size (69%) and velocity (57%) but low for tempo (35%) (Table 2). The common environment effect was highest for tempo (42%) and lowest for size (11%). Environmental influences unique to each twin explained a similar amount of variation for all three SITAR parameters.

## Discussion

In this exploration of the genetic architecture of early infant growth, we observed differences in the heritability of early and later weight, as well as between the three modeled growth parameters (size, tempo and velocity). Like weight at six months, size and velocity were highly heritable features of growth trajectories. However, tempo showed a smaller genetic effect and a stronger influence of the common environment. In the light of evidence that peak weight velocity may be an important determinant of later health, support for the influence of childhood environments provides a valuable starting point for investigations of the environmental determinants of infant growth.

Birth weight, weight at three months, and weight SDS change from birth to three months, all had low heritability in the present analysis, which is in line with previous studies [11], [12], [13], [14], [15], [16], [18]. Our results indicate that environmental variation in birth weight is predominantly owing to factors unique to each child. A Norwegian family-based study of birth weight observed a similar pattern, with estimates of 15% for the common *vs.* 32% for the unique environment effect [17]. Unique environmental influences may be higher for birth weight because placental factors affecting nutrient transfer (such as chorionicity, placental fusion and central vs. peripheral insertion of the umbilical cord) can create differences even between MZ twins who share the same genes and placenta [34], [35]. Accounting for unique variation in placental factors in twin analyses has been shown to increase the estimated heritability of birth weight [14].

Between birth and three months, growth rates change rapidly and therefore weight at a single time-point during this period may not reflect size as reliably as at later points when growth rates stabilize [36]. Lower reliability reduces estimates of heritability and common environment and inflates the unique environment effect, which could contribute to the apparent low heritability of weight in early life. However, the first three months of life has also been identified as a critical period when growth is nutrition dependent [37], and because nutrition is primarily an environmental exposure (albeit affected by infant appetite [38]), this could offer an alternative explanation for the low heritability for immediate postnatal growth; consistent with the moderate shared environment effect observed in this analysis.

By six months there was evidence for a stronger genetic influence on weight. Studies of human growth and body composition [39] indicate that immediate postnatal weight gain is predominantly composed of fat mass, but by six months, gain in lean mass makes a larger contribution, as indicated by increased height growth after six months [40]. Adult height is 80% heritable [41] and therefore if weight in later infancy has a greater contribution from height gain, then this may partly explain the rise in heritability observed during this period.

Modeling growth using all available weight measures using SITAR generates three variables that indicate individual variation from the average growth curve for the group. Heritability of the size parameter was 69%; similar to results for weight measures taken after 5 months [13], [18], [19] and to our estimate for weight SDS at six months. The higher heritability may also reflect improved reliability of ‘size’ (which summarizes many weights) compared with single measures of weight. Our estimate of 57% heritability for velocity was similar to the estimate for change in weight SDS from birth to six months in the present study, and also to velocity derived from a different model of growth in twins during the first two years [22], although higher than was found in a family-based study [24]. There is evidence that some obesity genes are associated with both weight (a proxy for size) and weight gain (a proxy for velocity) in early infancy [42], [43], [44]. Multivariate heritability models investigating the genetic correlation between size, velocity and tempo could add further insight into the physiological control of growth in infancy.

We are not aware of any previous studies that have examined the heritability of the timing of PWV (tempo in the SITAR model). Our results showed low heritability for tempo compared with size and velocity. Previous studies of the magnitude of PWV have shown associations with an earlier onset of puberty (a indicator of tempo in later childhood) [45], suggesting that infant and later childhood growth tempo are linked. Earlier maternal menarche has also been associated with more rapid growth in their infants; linking tempo across generations [46]. However, our estimate of heritability for tempo in infancy was lower than has been observed for the timing of puberty [47], [48], [49]; supporting different biological processes. The evidence that tempo is under primarily environmental influence is important in the light of evidence that growth and health risk are programmed [50]. It highlights the need for studies that directly assess the potential environmental exposures.

The present study is strengthened by the use of data from a large, population-based sample with multiple weight measures made by health professionals, which have been shown to be accurate compared with clinic measures [36]. SITAR modeling averages information across measurement occasions and therefore minimizes measurement error as well as summarizing growth over time, allowing for more reliable estimates. There are also limitations. Although the Gemini sample is reasonably representative of families in England and Wales [26], higher SES families are over-represented, as observed in other cohort studies [51], [52]. Twins also grow differently from singletons during infancy [53], although there is no evidence to indicate differences in the underlying physiology of growth. The twin method also makes several assumptions [54], e.g. that MZ and DZ twins have equal sharing of common environmental exposures, but even if the equal environments assumption is violated, the effect on heritability estimates is relatively small and unlikely to alter the qualitative conclusion that genetic influence on tempo is lower than on size or velocity. Zygosity was estimated using a validated parent-report questionnaire that has previously demonstrated 96% accuracy for classifying twins correctly compared with DNA testing [27].

### Conclusions

Using modeled growth parameters, we have shown that size and velocity are highly heritable whereas tempo (age at PWV) is largely explained by environmental factors. A better appreciation of the environmental determinants of infant growth will help inform the development of effective early interventions to promote healthy weight.
